# Mapping regenerative value network configurations of marine plastics in the global commons

**DOI:** 10.1007/s00267-025-02339-3

**Published:** 2025-12-10

**Authors:** Taryn Mead, Hanna Dijkstra, Kipp Godfrey

**Affiliations:** 1 School of Architecture and Design, IE University, C. Cardenal Zúñiga, 12 40003 Segovia, Spain; 2 Institute for Environmental Studies (IVM), Vrije Universiteit Amsterdam, De Boelelaan 1111, 1081 HV Amsterdam, the Netherlands; 3 School of Business, Western Colorado University, 1 Western Way, Gunnison, CO 81230 USA

**Keywords:** Regenerative value networks, Regenerative business, Marine plastics, Sustainability, Case studies

## Abstract

While many companies have made claims regarding regenerative efforts in recent years, analytical frameworks are sparse that clearly demonstrate how claims of regeneration can be differentiated from claims of sustainability. This study proposes the use of Regenerative Value Networks (RVNs) as a theoretical framework to analyze these claims and refine the descriptive approaches to claims of regenerative efforts, particularly for marine plastics in the global commons. In this exploratory study, five cases of new materials generated from marine plastics were analyzed to understand how value networks were constructed and what types of regenerative value were created. Data was collected through semi-structured interviews and thematic analysis was used in a comparative analysis across cases. Preliminary data analysis led to the question “But who is paying whom for what?” and a series of value configuration maps were developed to better understand these relationships. Types of value exchanged in the RVNs were categorized as material, social, ecological, and economic, contributing to a more robust theoretical framework for claims of regeneration in business. Further research should include quantitative measures of the regenerative impact of these value networks in the context of the global commons’ pollution, the role of certifications in regenerative business claims, and additional types of less tangible value created by RVN activities.

## Introduction

The scope of the modern ecological crisis far exceeds that first described as the ‘tragedy of the commons’ now nearly 200 years ago (Forester Lloyd, 1832; Hardin, 1968). In the original conceptualization, it was the *individual actions of extraction* that resulted in the tragedy for all stakeholders, leading to problems such as overfishing, deforestation, and drought. However, during our modern era of globalized material, supply, and waste management systems, a new type of tragedy has emerged in which the over-consumption of goods and services in one place then leads to a *collective accumulation of materials* in the global commons (Scheel, 2016). The last forty years of intensification of global trade have sent materials and pollution circumnavigating the planet, with many unintended global impacts (Christoff & Eckersley, 2013; Roome, 2011; Scheel, 2016; Young et al., 2006). The emergence of pollution that spans transnational boundaries, has created a new tragedy of the commons in the form of an excess of technical nutrients or pollutants (as defined by Braungart et al., 2007). These pollutants have no owner, nor have national or international regulatory frameworks been successful at reigning in the global impacts of the pollutants, despite widespread public outcry (Vince & Hardesty, 2018).

The need for regeneration in socioecological systems is increasingly evident not only to prevent further tragedy of the commons, but to remediate the damages already done to the environment and return to a ‘healthy state’ (Morseletto, 2020). While issues in the commons are typically governed by government entities and organizations with international influence, the issue of marine plastic pollution remains in an ambiguous realm of policy, pollution mitigation, and enforcement. However, there has been a private sector response to the increase of transnational pollutants through efforts to prevent, collect, transform, and monitor pollution (Dijkstra et al., 2021). Applying systems thinking in their business model development, many companies are adapting the role of waste in their value networks to view it as a valuable material in their supply chain (Perey et al., 2018) and applying a regenerative approach to the issue of plastics pollution.

Across sectors, businesses are applying regenerative theory to their activities and differentiating it from their existing sustainability and circularity agendas (Caldera et al., 2022; Das & Bocken, 2024; Hahn & Tampe, 2021; Konietzko et al., 2023; Morseletto, 2020; Salonen et al., 2025; Verkooijen & Janssen, n.d.). In Konietzko’s (2023) review of claims of regenerative business models, there are key differences between sustainable, circular, and regenerative business practices. Sustainable business models focus on reducing the negative impact and harm done by business. Circular business models aim to make business as self-sustaining and resource efficient as possible through closed and slowed resource loops (Bocken et al., 2016; Bocken & Ritala, 2022; Konietzko et al., 2023). Regenerative business models place economic activity within the context of ecological and social systems and focus holistically on improving the health and prosperity of ecological systems through economic activities (Das & Bocken, 2024; Hahn & Tampe, 2021; Konietzko et al., 2023).

Many private companies are applying regenerative principles to their business operations and strategies creating complex relationships of value exchange (Das & Bocken, 2024; Gordon et al., 2023; Konietzko et al., 2023; Paolini et al., 2024). Considering businesses focused on plastic pollution, specific questions arise about what is externalized waste, what is raw material, and how and for whom economic benefits are created (Perey et al., 2018). With few exceptions (e.g., Gualandris et al., 2024), existing studies related to regenerative business have analyzed publicly available narratives related to the regenerative benefits of various case studies (e.g., Konietzko, Das and Bocken, 2023; Das and Bocken, 2024) or offered broad theoretical framing without empirical analysis (e.g, (Hahn & Tampe, 2021; Salonen et al., 2025)). However, none of these studies describe the rich, nuanced regenerative relational value exchange in detail that would guide potential practitioners in how to create regenerative value. Without clarifying models and frameworks, there is a risk of greenwashing and the conflation of sustainability, circularity, and regeneration (Das & Bocken, 2024). The value networks that enable innovation for regeneration are expanding globally with varying degrees of regulation and incentive across industries and material categories and yet, there is no unifying framework that identifies regenerative value creation in a practical and applicable way, differentiating and cataloging the types of regenerative value that can be created.

This study aims to address this gap through a series of case studies and a comparative case study analysis that describes the specific relational mechanisms through which RVNs can positively contribute to nature and society (e.g., Jain & Gualandris, 2023; Kennedy & Linnenluecke, 2022). As interview data collection in this study progressed, the diversity of ways that these actors relate to one another led to the question “but who is paying whom for what?” This study identifies the relationships and incentives that enable complex webs of intermediary remanufacturers, informal waste collectors, community and nonprofit stakeholders, technical partners, and brands to create RVNs in the context of plastic pollution mitigation.

## Literature review

Though research in the area of regenerative business practices has emerged relatively recently, there is a robust interdisciplinary theoretical foundation through which to analyze the cases. The interviews and issues are explored through the lenses of regeneration of the global commons, waste valorization, and value network creation.

### Regeneration in the global commons

Although still widely contested, the concept of regeneration has been well-described across many disciplines (Paolini et al., 2024; Salonen et al., 2025). Grounded in place-based and indigenous philosohies, it asserts that human-designed systems should align with the self-sustaining principles of whole living systems (Hecker & Toivonen, 2024; Mang & Reed, 2012; Reed, 2007). The regenerative perspective views earth-human interactions as proceses of symbiosis and co-evolution requiring fully apperceptive engagement in socio-ecological systems (i.e., apperceptive meaning conscious of participation, which is recognized and understood) (Albrecht, 2020; Braungart & McDonough, 2009; Hecker & Toivonen, 2024; Wahl, 2016). Through the lens of organizational theory, Konietzko et al., (2023) identified the following three characteristics of regenerative organizations: “1. Recognize that human societies are deeply embedded in the biosphere, and that they depend on the health of the biosphere for their own health; 2. Have a value proposition of planetary health and societal wellbeing to nature and society at large; 3. Give more than they take and strive for net positive impact” (Konietzko et al., 2023, p.384). Similarly in the innovation literature, Mead (2018) defined *regenerative innovation* as “Innovation strategies that create infrastructure to utilize a pollutant and source of socioecological harm currently present in the global commons to create novel value for the organization and develop inclusive social capital while reducing ecological damage” (p.245). Despite the risks of greenwashing, the flexibility of the definition of regeneration has enabled its use across disciplinary boundaries, giving it strength as a framework for collaborative social agreements (Paolini et al., 2024). For the purposes of this study, we consider regenerative theory through a combination of disciplinary lenses as described above, focusing on the social, ecological, material, and economic implications of each case study.

### The valorization of waste in the global commons

For most common waste streams, the ownership and responsibility of waste materials has shifted through time from local governmental management to privatization. However, the ownership of materials in the global economy is becoming increasingly fluid as the value of these materials changes in response to global demand and policy initiatives for more sustainable systems of production and consumption (Velis & Vrancken, 2015). Recent changes in international policy, such as the restriction of imports of plastic, electronic, and textile waste, have increased the global dialogue on the valorization of waste materials more broadly and brought to the forefront the regional diversity of waste management systems and involved stakeholders (Seay, 2020).

Among many local and global solutions, the re-valorization of plastic waste has been proposed as a potential solution to the plastics pollution problem (Ten Brink et al., 2018), and these solutions must be low cost, socially acceptable, financially viable, and ecologically sensitive, including stakeholders in the regions where plastics pollution is the most widespread (Joshi et al., 2019). In many parts of the world, bottom-up informal initiatives create entrepreneurial opportunities around capturing, processing, and recycling waste (Anabaraonye et al., 2022; Gall et al., 2020; Mihai et al., 2022). These ventures are highly adapted to localized contexts and contribute to waste remediation that otherwise would not occur (Grassin & Dijkstra, 2024). Frequently initiated by ‘transition-oriented entrepreneurs’ (Dijkstra & Planko, 2023) and corporate intrapreneurs (i.e., entrepreneurs who innovate within an organization), they seek to meet the material needs of their business through the use of waste leveraging the workforce of informal waste collectors. While not central to this study, it should be noted that the role of informal waste collectors and recyclers is difficult to quantify, track, and incentivize, leading to a lack of quality data for the role that this activity plays in the global commons and economy (Scheinberg et al., 2016).

Bollier (2006) suggests that new forms of collaboration are creating a ‘commons paradigm’ in which economic activities are developed collectively to meet social, cultural, physical, and economic needs based not solely on competitive mechanisms, but rather on shared values such as reciprocity and democratic exchange of value. Ventures emerge in response to entrepreneurial opportunities, such as collecting or recycling waste, delivering waste management services, or creating novel products and services. In doing so, these inititatives reduce environmental damages, deliver social benefits, and capture economic value. In this context of pollution in the global commons, this self-organization and access to waste materials are the driving mechanisms of RVNs as demonstrated by the cases in this study.

### Regenerative value networks as a novel theoretical framework

Building on concepts of circularity and sustainability, the literature related to regenerative organizations and business models has gathered momentum in recent years from an array of perspectives in the management literature (e.g., Bocken et al., 2016; Lüdeke-Freund et al., 2018; Mead, 2018; Muñoz and Branzei, 2021; Konietzko, Das and Bocken, 2023; Drupsteen and Wakkee, 2024). While a few case study collections and theoretical descriptions have been created, the specific mechanisms by which different forms of regenerative value are created and by which stakeholders, have not yet been described in nuanced detail. The exact mechanisms of value exchange and creation are only discussed at a high level, emphasizing new business model development as a primary driver rather than the more holistic view of regeneration. Somewhat problematically, this positioning puts the onus of the burden of regeneration on start-up firms, which generally have little expertise in the development and maintenance of supply chain complexity, especially at a scale needed to address the goal of mitigating plastic pollution in the commons. To address problems of this scale, theoretical frameworks are needed which communicate across public, private, and NGO stakeholders to generate new global initiatives in pollution mitigation.

In recent decades, the concept of ‘value networks’ has emerged guided by the concepts of sustainability, globalization, collaboration, intangible assets, flexibility, and agility (Ricciotti, 2020). As an evolution of value chains which rely on a more top-down, economic-driven approach to sourcing, the value networks framework recognize that the process of supplying materials for systems of production and consumption is rarely a linear one. Rather it is more relational, with inputs from a web of producers, suppliers, and manufacturers. For instance, as Brown and Bajada (2018) have summarized in the context of circular supply networks, “Cooperation and collaboration of the multiple stakeholders in circular supply networks is fundamental and necessary for change in sustainable practices to occur at both the organization and network levels. These stakeholder networks include investors, legislators, government agencies, special interest groups, media, environmentalists, manufacturers, and retailers” (p.644). Regenerative value logics have also been described in recent years, highlighting how local spatiotemporal conditions align with life-supporting ecosystem flows and participatory, decentralized, and collaborative governance mechanisms reflect historical and social contexts (Casadiego et al, 2023). Existing frameworks in regenerative business are insufficient to clearly articulate the specific policy mechanisms, international collaborations, and informal entrepreneurship that might enable this type of innovation.

In this study, we define Regenerative Value Networks (RVN) as the complex integration of processes, resources, and stakeholders engaged in the regeneration of socioecological systems through the development of novel forms of material, social, ecological, and economic value. The forms of value are defined as follows:

- Material value created through physical resources, products, and material flows.
- Social value generated by strengthening relationships, collaboration, opportunity, and community well-being.
- Ecological value arising from removal of pollution and restoring ecosystems and biodiversity.
- Economic value measured through financial viability, trade, and monetary resource circulation.

The different types of value (material, economic, ecological and social) are being created within a RVN and can thus be conceptualized as elements of regenerative value. RVN stakeholders are those that participate in value exchanges including traditional (i.e., manufacturers, brands, suppliers, etc.) and unconventional (i.e., informal waste collectors, NGOs, community banks, etc.) partners, through webs of value exchange. RVNs emerge through the mutually beneficial interactions between the four types of value and their respective stakeholders. The framework is used to analyze in-depth case studies in which internal stakeholders describe how they built RVNs for their businesses through exchanges of various types of value. The analysis then aims to demonstrate examples of how RVNs are created through the efforts of a singular firm of focus so that the cases may serve as models for future entrepreneurs and innovators. The RVN framework can be used to analyze existing regenerative business claims and further define how regenerative business can be developed.

## Methods

Given the relatively small number of companies claiming RVN activities, and the still exploratory stage of research in this area, a qualitative methodology was applied. Results were derived through a multi-case study analysis which compares and contrasts the cases (Yin, 2009) on specific questions related to value exchange and monetization which emerged through an iterative, inductive research process (Yin, 2009). The cases were selected via purposive sampling due to the exploratory aims of the study. The cases represent companies and partners engaging in RVN with plastics, who were readily accessible and relevant to the study context. The data supporting the case study analysis was gathered through interviews and public document analysis

As described by Shackleton et al., (2022), the interview method is appropriate to understand the socio-ecological, institutional, and social-relational dimensions of socioecological systems and as such, was the selected data collection method. The aim of the interviews was to gather diverse, open-ended insights into emerging themes rather than test predefined hypotheses about the networks. In-depth, semi-structured interviews (Shackleton et al., 2022) were conducted with various stakeholders including brand representatives, materials experts, nonprofit partners, entrepreneurs, and intermediary suppliers. Informed consent forms were delivered via email before each interview and agreed upon either by signing or verbally during the recorded web-based video interviews. Details of stakeholders interviewed are available in Table 1. Research questions are available in Appendix A and full interview data are available upon request.

**Table 1:** RVN Case Studies

Case #	Case Name	RVN Partners (Representatives interviewed are bold)	Materials Collected	Region of Material Collection	Value Added Product(s)
1	Ocean Bottle®	**Ecover (2 – Innovation Manager & Management Consultant), Logoplaste (1- Design Consultant)**, and Plastic Whale	Ocean and Ocean-Bound Plastics	Europe	Consumer dish soap bottles
2	Net Your Problem	**Grundens (1 – Marketing Manager), Net Your Problem (1 - Founder)**, Rugged Seas, Odyssey Innovation, Waterhaul, other product brands, local non-profit and collection partners, and numerous other land management agencies.	Fishing Nets and Other Commercial Fishing Equipment	North America	Recycled pellets and yarns
3	Stoked Plastics®	**Opolis Optics (1 - Founder)**, Materials Research Partner, Non-profit clean-up partners	Plastic Water Bottles, Ocean-Bound Plastics	Southeast Asia and Africa	Sunglasses and rPET
4	Netplus®	**Bureo (3-Founders), Patagonia (1-Sustainable Materials Specialist)**, Jenga, Costa, and numerous other product brands and local non-profit and collection partners	Fishing Nets	Latin America	Skateboards, games, and other consumer goods including textiles
5	Net-Works®	**Interface (5 – Chief Innovation Officer, Project Managers, Sustainability Directors)**, Aquafil, **London Zoological Society (1- Project Manager)**, and numerous other nonprofit partners	Fishing Nets, Carpets, Other sources of Nylon-6	Southeast Asia and Africa	Carpets

Video recordings were transcribed and descriptive coding methods were used (Fereday & Muir-Cochrane, 2006) to inductively generate thematic categories of analysis (see Table 2 for emergent themes used in cross-case analysis). Interview data were manually coded using an iterative process involving multiple researchers who independently applied and refined a shared coding scheme to improve reliability without the use of coding software. Given that cases exemplify “multiple places/sites around the world”, “transformation”, and “collective action and collaborative governance” (Pahl-Wostl et al., 2022), the coded data were then used to create descriptive case analyses. Some of the cases have existing documentation in the academic literature and popular media and in these cases, publicly available documentation was incorporated into the case descriptions to add additional detail and nuanced description. Though condensed for the purposes of publication, descriptive case studies created a nuanced and detailed understanding of the phenomenon in real world contexts (Corbin & Strauss, 2015). For all cases, value configuration maps were created to describe the value exchange between the stakeholders and conduct cross-case comparisons between value configurations (Brehmer et al., 2018), from the vantage point of the focal material and its respective company. The visualization of the nuanced value exchange of each case was addressed with flow diagram mapping material, social, ecological and economic exchange to reduce the narrative complexity of each case.

**Table 2:** Themes Used In Comparative Case Analysis of Interview Data

Partners	Strategy
Startups	Market forces
Established Brands	Partnerships
NGOs	Ad-hoc business planning
Informal waste sector	Incubator/accelerator programs
Community stakeholders	Future plans
	Venture capital
**Value Exchange**	Scalability
Value-added product	Competition with virgin materials
Supply chains	
Product development	**Value**
Motivation for starting the company	Value exchange models
	·Where, how, who creates valorization
**Policy**	Types of value - Material, Economic, Social, Ecological
Policy incentives needed	Who “owns” or “values” the materials
Policies that inhibit progress	Who is paying whom for what?
International waste trade	
Certifiation and standards	

## Results

The five RVN case studies are summarized in Table 1 and the following section describes each case in detail. Each case description highlights regenerative activities in *italics* and includes a figure demonstrating value exchange among key stakeholders. Presented in order of increasing complexity, the cases illustrate how material, social, ecological, and economic value are generated and exchanged in RVN, leading to *material, social, ecological and economic regeneration*. Finally, Table 3 provides a detailed comparative analysis of the regenerative value exchange across cases.

**Table 3:** Cross Case Comparison of Types of Regenerative Value Created

Case	Material Regeneration	Economic Regeneration	Ecological Regeneration	Social Regeneration
1. Ocean Bottle®	Ocean plastics and sugarcane based bioplastics converted into bottles for liquid soap.	Circulating corporate innovation funds to support biomimetic designers and nonprofit organizations.	Removing ocean plastic pollution from waterways and beaches	Partnerships with fisherman, school groups, nonprofit stakeholders
2. Net Your Problem	Fishing tackle and gear, Fishing nets converted into upcycled tote bags, sunglasses, kayaks, and watersports gear.	Creating new value from discarded industrial materials which would otherwise not be recovered. Creating new opportunities for entrepreneurs.	Recovering fishing equipment which otherwise lacks adequate disposal options and removing it from ecological systems.	Engaging fishers in the active regeneration of community spaces through clean-up efforts.
3. Stoked Plastics®	Plastic bottles, specific type, converted into sunglasses and ski goggles.	Creating new value for plastic waste in coastal communities with limited economic opportunity.	Recovering ocean-bound and ocean plastic from coastal communities in lesser developed economic communities.	Engaging impoverished communities in areas prone to develop terrorist activities with new forms of social engagement.
4. Netplus®	Ocean plastic, ocean-bound plastic, discarded fishing nets collected and recycled. Fishing nets re-polymerized into Nylon-6 and re-manufactured in apparel and outerwear.	Creates new jobs and new source of revenue for fishers and community members in rural areas.	Removal of discarded nets from marine and coastal ecosystems. Revenues fund various community-based ecological restoration projects and an animal rehabilitation center.	Community projects funded in schools and communities for plastic clean-up, wastewater treatment, and local food production.
5. Econyl®	Discarded fishing nets and used carpet re-polymerized into Nylon-6 fiber, then remanufactured into carpet and textiles used in apparel.	Creates a source of revenue for community members, established a community banking system, and enabled micro-loans which supported economic development. Post-natural disaster, community banking aided in faster reconstruction timelines.	Removal of discarded nets from marine and coastal ecosystems	Community members created a new grassroots banking system, empowering investment in education and workforce development.

### Case 1: Ocean Bottle®

**Fig. 1:**
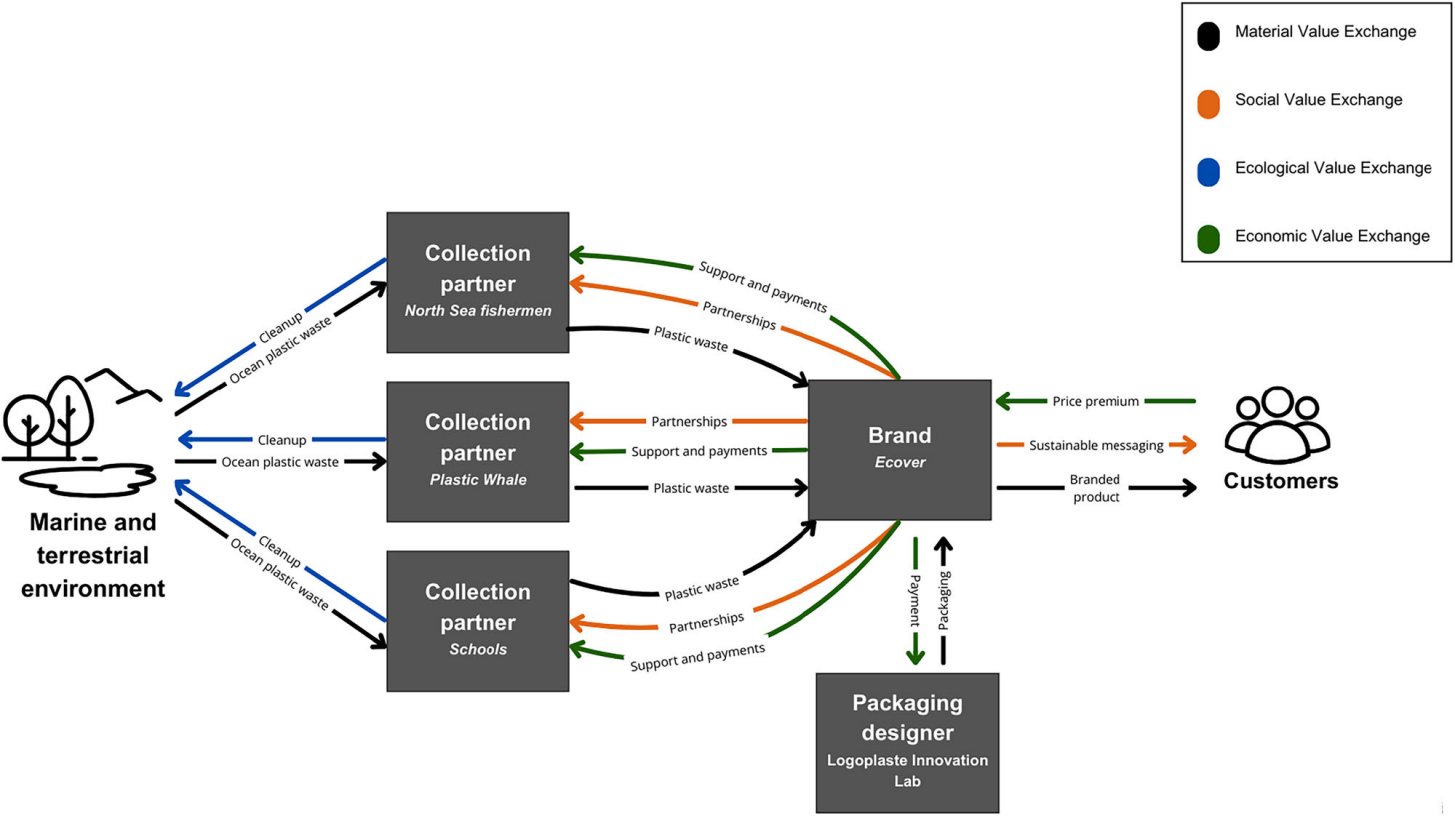
Regenerative Value Exchange of Case 1: Ocean Bottle®

After Interface’s Net-Works product line became public in 2012 (see Case 5: Econyl®), leaders within the design unit at Ecover, a multi-national corporation selling cleaning products, were interested to develop a similar product that embodied the principles of regenerative design. They sought to re-assimilate waste materials from ecological systems, meeting their requirements for raw materials while improving ecological health. The outcomes of this initiative resulted in the “Ocean Bottle” which demonstrates *material regeneration* by remanufacturing collected ocean plastic into raw materials. In partnership with the Logoplaste Innovation Lab, a research and design firm, Ecover contracted a biomimicry-trained packaging designer to develop a bio-inspired bottle design optimized for strength and weight. They also formed partnerships with North Sea fishermen, schools in the Netherlands, and the ocean plastic clean-up organization Plastic Whale, forming new collaborative alliances between a diversity of stakeholders to source ocean plastic and develop new pathways for volunteer engagement socioecological systems *(social regeneration)*. *Social regeneration* is also enabled through consumer education in the package labeling and product messaging. This initiative supported *economic regeneration* by creating jobs and new income streams through the collection of litter and waste. Starting with just 10% ocean plastic in its first production run in 2014, by 2017, the Ocean Bottle contained 50% ocean plastic and 50% recycled plastic (Nolan, 2018). From 2014-2018, they produced 425,000 bottles using collected ocean plastic, now incorporating ocean plastic from Brazil in addition to that of Northern Europe (Nolan, 2018). Removing this large quantity of plastic debris from the ocean ecosystems through a geographically diverse collection strategy suggests an effort to globalize the *ecologically regenerative* impact of the initiative. Recent material trials have also incorporated biodegradable plastics sourced from sugar cane, further reducing petroleum-based plastics (Nolan, 2018). See Fig. 1.

### Case 2: Net Your Problem

The founder of Net Your Problem worked as a North Pacific fisheries observer from 2010 to 2015, during which time she identified the persistent issue of waste generated by the commercial fishing sector. On remote islands with economies dependent on fishing, the disposal of obsolete fishing gear presents logistical and infrastructural challenges. In response, Net Your Problem was established in 2020 to develop new forms of value creation with these waste materials. At the time of interview, the organization operated as a small for-profit business with a staff of four based in the United States.

An early partnership with Grunden’s, a company producing outerwear for commercial fishers, provided initial financial support in the form of a sponsorship. Additional brand collaborations have included Rugged Seas, Odyssey Innovation, and Waterhaul. As of spring 2024, Net Your Problem reported new partnerships with private-sector firms, nonprofit organizations, and academic institutions in the southeastern United States. Out of necessity, the organization maintains a diversified financial model that includes marketing sponsorships, government contracts for material disposal, agreements with port authorities and industry groups, and sales of recovered materials to recyclers that convert fishing nets into raw plastic and nylon feedstock.

**Fig. 2:**
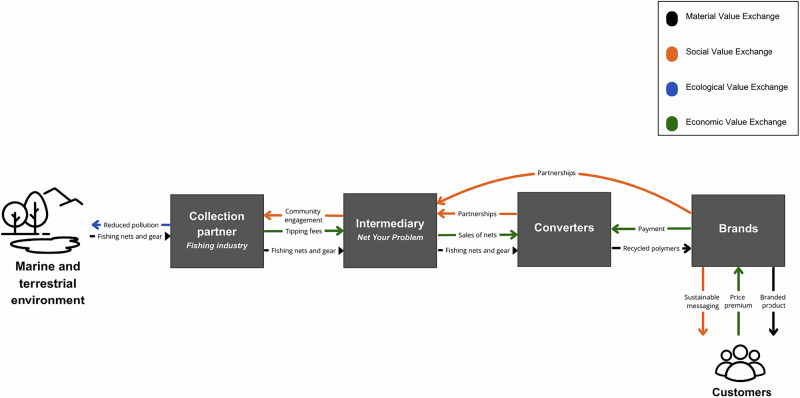
Regenerative Value Exchange of Case 2: Net Your Problem

Net Your Problem engages a range of stakeholders, including industry associations, fishing fleet owners and managers, recyclers, net manufacturers, and government agencies, to facilitate the *material regeneration* of fishing gear through recycling, upcycling, and remanufacturing. The collected materials are remanufactured into upcycled goods and new materials through pelletization and repolymerization. Given that much of the waste in this case study is currently held in private shipyards and adjacent public lands, the active engagement in cleanup efforts is a substantial move towards community engagement and participation in pro-environmental behaviors in a traditionally resource extractive industry, contributing to *social regeneration. Economic regeneration* is created through defined revenue streams such as fee-for-service contracts, corporate sponsorships, and the commercial sale of processed materials, circulating funds through various small businesses and providing materials for new product startup companies. In parallel, the removal of waste from marine environments supports *ecological regeneration* by mitigating the impacts of discarded gear such as ghost fishing, and in their coastal collection sites, supporting the recolonization of coastal plant and animal species, and restoring the natural geomorphology of the landscape. See Fig. 2.

### Case 3: Stoked Plastics®

Opolis Optics is an ocean plastic material company founded in 2020 in Southeast Asia. The founder had previously worked as a USAID contractor focused on anti-terrorism initiatives in impoverished regions. This experience shaped his recognition that economic deprivation and limited livelihood opportunities can drive participation in criminalized activities. In response, he began to explore alternative models for economic engagement in regions where poverty and plastic pollution intersect, leading to collection efforts in Kenya, Indonesia, and the Philippines, with a focus on plastic bottles due to their visibility and recyclability.

Opolis Optics provides compensation for plastic bottle collection through collaborations with local community partners, including informal waste collectors, as a sourcing strategy for marine plastic. These efforts contribute to *social regeneration* by introducing the sale of collected recyclable plastics as an income-generating alternative to engagement in illicit economies. *Ecological regeneration* is addressed through the removal of plastic waste from local ecosystems, cleaning of waterways, and reduction of pollution, and *material regeneration* is realized through the transformation of collected plastics into usable polymers for manufacturing eyewear products such as sunglasses and ski goggles.

**Fig. 3:**
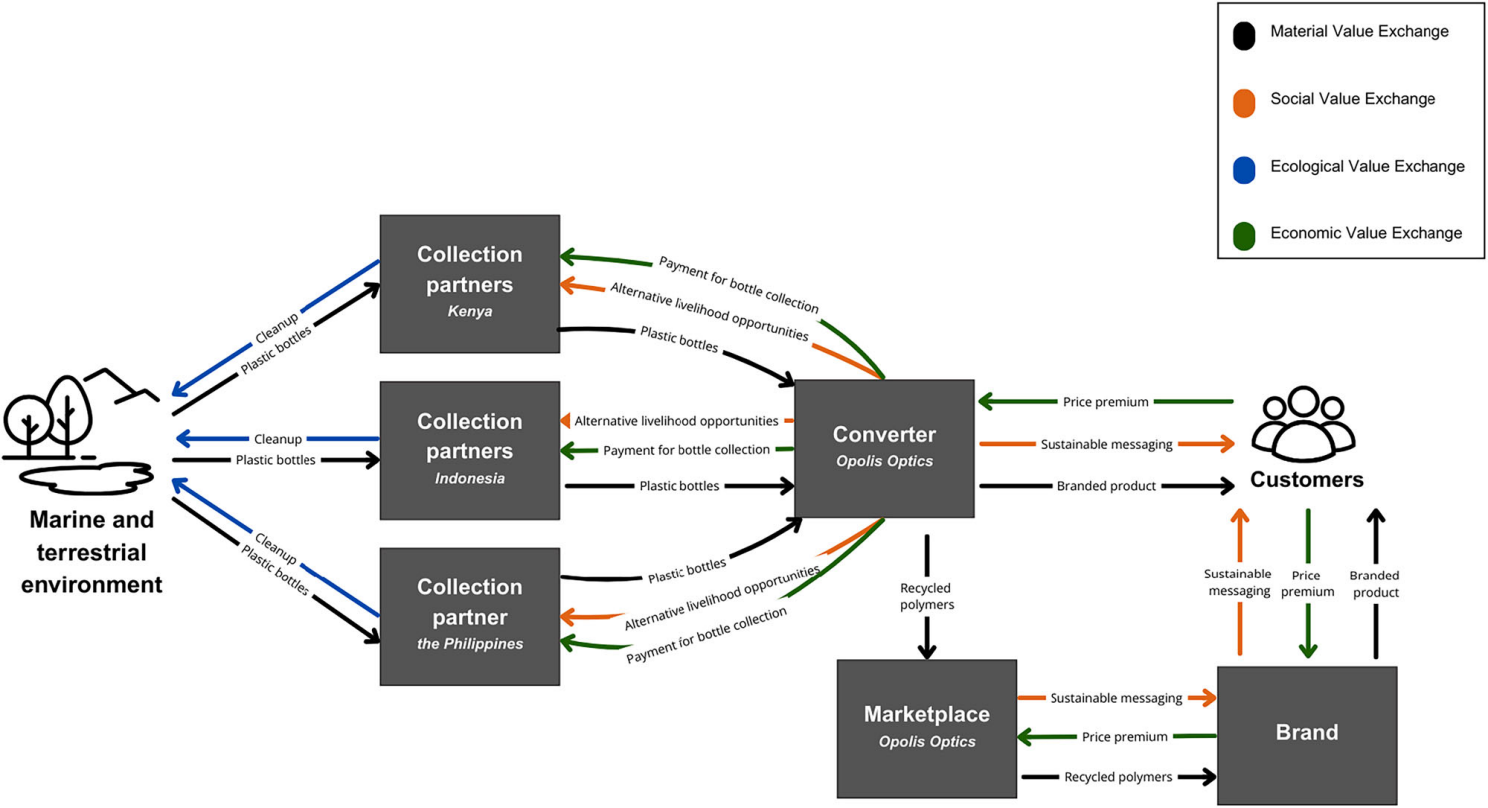
Regenerative Value Exchange of Case 4: Stoked Plastics®

Initial production efforts operated at a small scale, utilizing ocean and ocean-bound plastic to create eyewear. As product development progressed, Opolis partnered with biomaterials and plastics researchers to formulate additional polymer blends to enhance the strength and durability of their materials. This process expanded the material utility of collected plastic waste and enabled the creation of new value-added products. Subsequently, the company launched a proprietary certified material, Stoked Plastics®, with the objective of becoming a value network intermediary for other manufacturers. This involved marketing recycled plastic pellets to external firms which diversified their revenue streams. At the time of writing, Opolis was in the process of commercializing this pelletized material and seeking partnerships with brands to support its integration into broader manufacturing systems. This approach exemplifies *economic regeneration* through the development of new value streams and business models grounded in *material regeneration.* See Fig. 3.

### Case 4: Netplus®

Bureo was founded in 2013 by three long-standing friends with backgrounds in surfing and ocean conservation who identified an entrepreneurial opportunity to address ocean pollution. Early-stage funding from StartUp Chile supported their initial development of partnerships with fishermen and informal waste collectors in coastal fishing villages in Chile. These collaborations facilitated the collection, cleaning, and pelletizing of discarded fishing nets composed of Nylon-6. This process exemplifies *material regeneration*, transforming marine plastic waste into new consumer products, with skateboard decks serving as their initial market offering through direct-to-consumer sales.

To expand their value network, Bureo hired additional sourcing managers who strengthened relationships with fishing communities and industry associations to better develop the social networks needed to collect discarded fishing nets and keep future net waste from being discarded. These place-sensitive collaborations contributed to the formalization of informal waste management systems and scaling of collection across other parts of South and Central America. This approach to *social regeneration* engages and enables local fishing communities by creating alternative economic pathways in waste recovery and maintains their connection to the value network partners throughout the net’s entire life cycle.

Bureo also entered material supply partnerships with consumer brands to distribute their recycled nylon pellets. One pivotal partnership involved Patagonia’s venture capital organization, which supported the acquisition of manufacturing equipment, expanding Bureo’s processing capacity and enabling application across a wider range of products, including textiles. Beyond manufacturing, Bureo partnered with nonprofit and educational organizations to raise awareness of ocean plastics through programming, clean-up initiatives, and collaborative outreach efforts, further enhancing *social regeneration*.

**Fig. 4:**
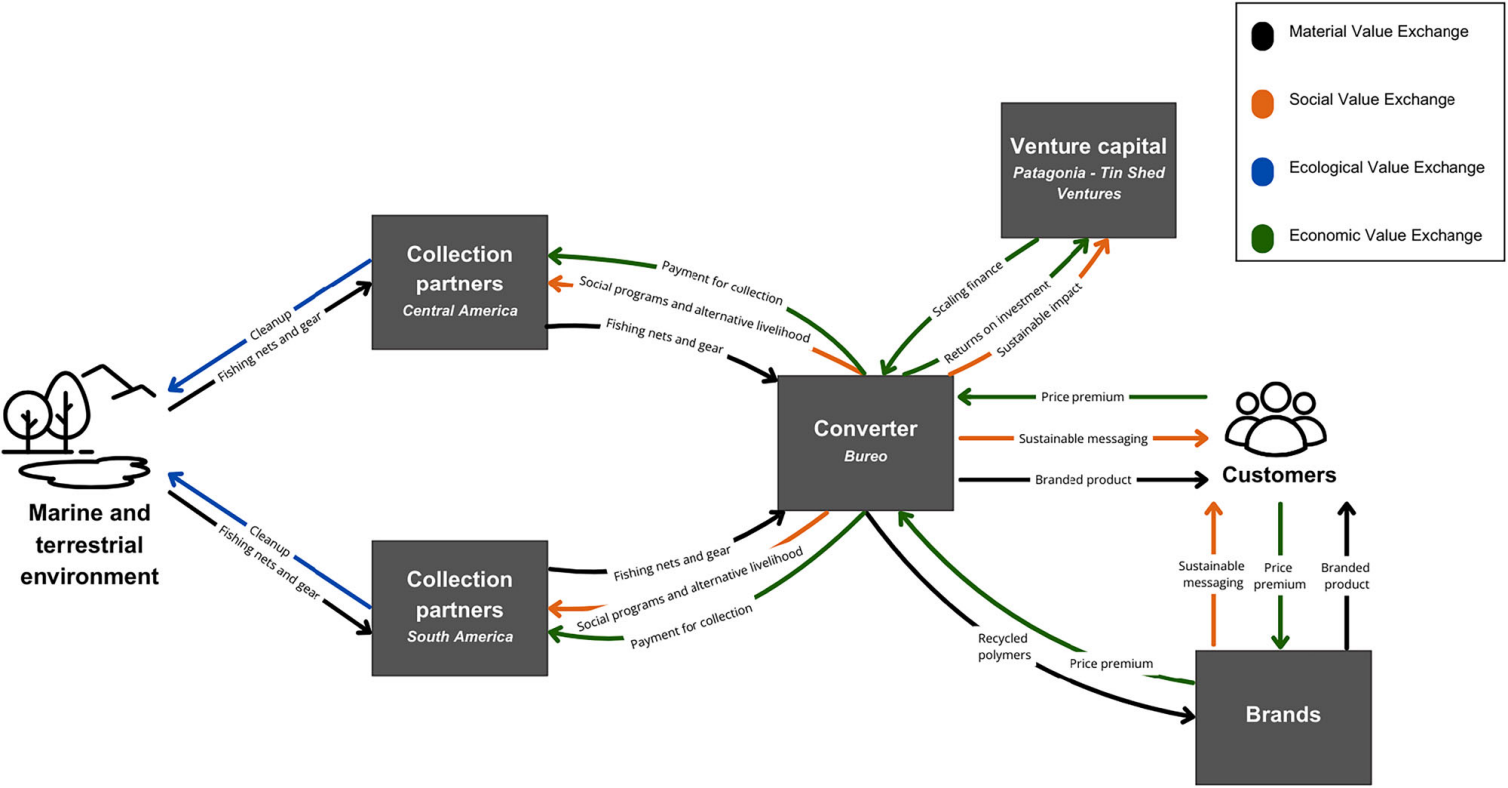
Regenerative Value Exchange of Case 4: Netplus®

As the organization evolved, Bureo transitioned from a vertically integrated, consumer-facing brand toward a business-to-business model. Through the development and sale of its proprietary raw material NetPlus®, the company shifted focus to becoming a material supplier for other manufacturers. This strategic pivot enabled operational streamlining and an emphasis on expanding their marine waste recovery efforts and reintegration of recycled materials into industrial supply chains (Mead & DeMerchant, 2024). Through these activities, Bureo contributes to *ecological regeneration* by mitigating and preventing pollution in marine ecosystems due to discarded commercial waste in these communities and broadening the infrastructure for marine plastic collection. The effort also advances *economic regeneration* through the creation of value for waste materials, diversifying employment opportunities in coastal communities, and empowering local actors in new forms of revenue generation. See Fig. 4.

### Case 5: **Econyl®**

In 2012, Interface, a carpet and textile company based in Georgia, USA, began exploring additional sources of recycled nylon for integration into its supply network. Having been an early adopter of sustainable business practices since the 1990s, the company had committed to long-term goals of emissions reduction and environmental impact mitigation. These efforts included the development of proprietary carpet recycling technologies and early engagement in policy initiatives related to carpet recycling in the United States. As the availability of recycled Nylon-6 from existing sources became insufficient to meet production demands, Interface initiated a search for alternative sources of post-consumer nylon fiber.

Identifying fishing nets as a potential source, Interface collaborated with the London Zoological Society to assess the feasibility of recovering discarded fishing gear, including ghost nets, for reintroduction into their supply chain. Through partnerships with nonprofit organizations and informal waste collectors, including Southern Partners, and Fair-Trade Corporation, community-based collection sites were established in the Philippines and Cameroon (Hower, 2016). Simultaneously, Interface worked with its supplier Aquafil to develop remanufacturing technologies capable of depolymerizing recovered nets into new nylon fiber, branded as Econyl®, for use in carpet and textile production. These efforts lead to *material regeneration* with increased collection of waste and enhanced technical pathways for recycling.

To facilitate net collection efforts, the initiative developed foundational financial infrastructure within participating communities specifically through the establishment of community banking systems, microfinance opportunities, and credit programs. These mechanisms enabled local individuals, many of whom previously had limited or no access to formal financial services, to receive direct compensation for the nets they collected. Importantly, these payments were not merely transactional; they catalyzed localized economic activity, allowing participants to reinvest earnings into small-scale enterprises, pay school fees, purchase essential goods, or save for future needs (Luqmani et al., 2017). This redirection of capital back into the local economy illustrates *economic regeneration*, where previously undervalued or discarded materials (e.g., marine debris) are transformed into a source of financial security and opportunity.

First piloted in a fishing village in the Philippines, the initiative supported the emergence of decentralized, community-led financial systems explicitly tied to environmental stewardship and marine debris recovery. By integrating economic incentives with conservation goals, the program restructured the relationship between local residents and their surrounding ecosystems by treating waste not as a nuisance but as a resource that could generate livelihood benefits. Branded as Net-Works® by Interface, the program exemplifies *social regeneration* in several key ways. It strengthened community agency by giving residents greater control over both financial and environmental decision-making processes. It also fostered inclusive participation by actively engaging women, informal workers, and other marginalized groups in meaningful roles within the value network. Additionally, the program contributed to social cohesion by encouraging collective environmental action and reinforcing a shared sense of responsibility for local marine stewardship.

**Fig. 5:**
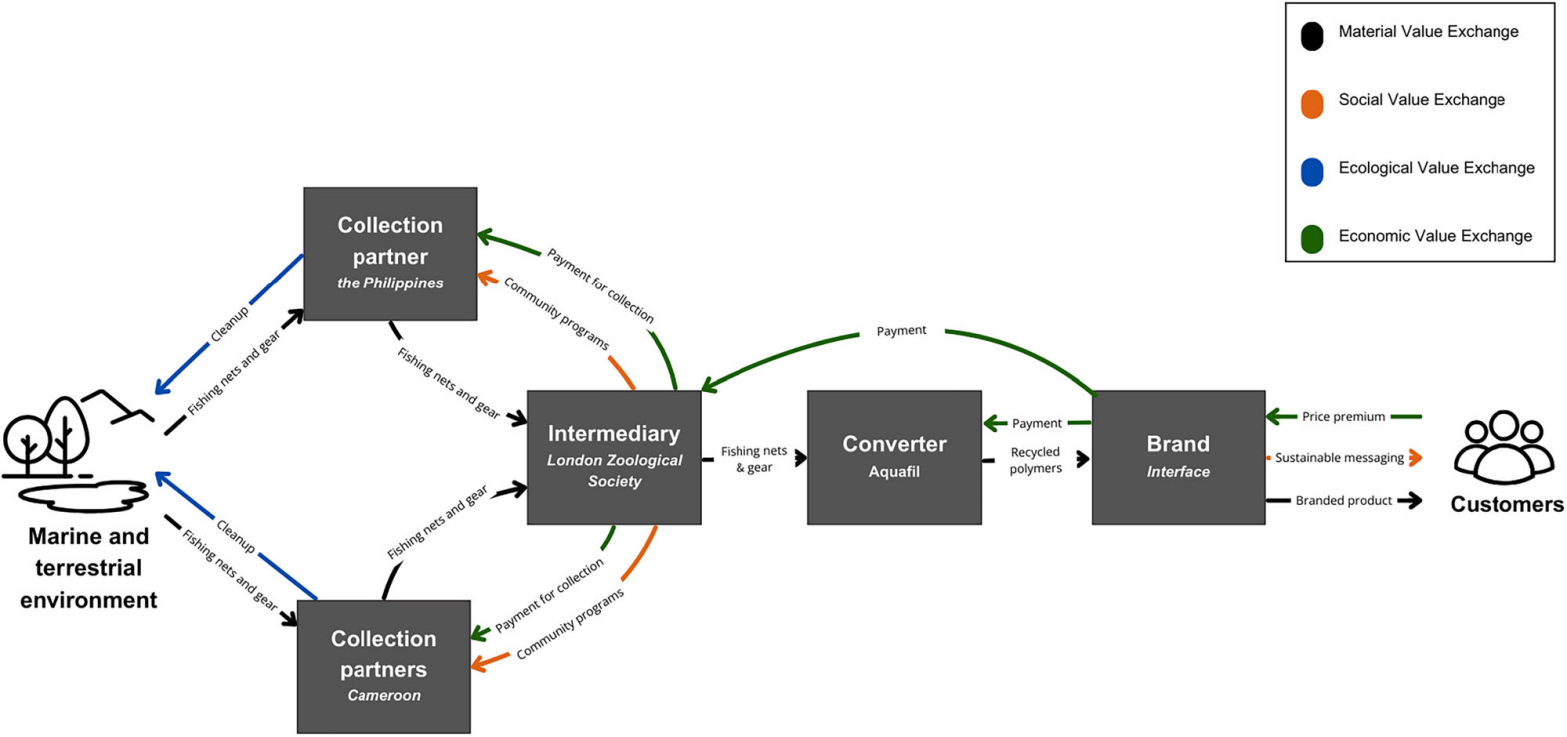
Regenerative Value Exchange of Case 5: Econyl®

Interface continues to source Econyl® yarn through this collaborative network, a case of *material regeneration* in which discarded marine plastic is converted into industrial feedstock for new textile products. Concurrently, the extraction of ghost nets from marine environments contributes to *ecological regeneration* by reducing threats to marine and coastal life, allowing ecological restoration of coastal habitats and the return of the natural circulation of sediments to beaches and waterfronts. Collectively, these efforts illustrate the formation of a regenerative supply network that integrates community-based economic activity with the technical requirements of industrial recycling (Hower, 2016; Luqmani et al., 2017). Interviewees in this case frequently questioned how they could “create conditions conducive to life”, one of the principles of biomimicry that they have been engaged with since the mid-1990s. See Fig. 5.

### **Cross case comparison**

Table 3 briefly summarizes the value exchange mechanisms from each case to demonstrate similarities and differences between the five case studies. Each of the five cases illustrates how new sources of regenerative value are created through the networks that they develop in their entrepreneurial and sourcing activities. While each case is unique, there is a general pattern of for-profit companies generating income through the sale of re-manufactured materials to fund or directly create social and ecological impact through their business operations. The materials created by these companies re-assimilate wastes from various countries and the global commons into material supply chains for products that would otherwise be produced with virgin petroleum polymers.

## Discussion

This discussion section first provides a general overview of *RVN creation*, the process through which RVNs generate new forms of value from polluting materials. It then highlights a few thematic insights that emerged from the interviews and case study analysis, including topics of branding and positioning, governance and accountability, scaling and systems change, limitations, and future research.

### Overview of RVN creation

The following overview, based on case narratives, outlines the common patterns driving the formation of RVNs. As with other global commons pollutants (e.g., greenhouse gas emissions) widespread public concern about plastic pollution has spurred a plethora of private-sector initiatives aimed at turning pro-environmental consumer preferences into business opportunities. While international policy responses have lagged (Dijkstra et al., 2022), both start-ups and established companies across sectors have recognized consumer demand for alternatives to virgin plastic and responded by developing regenerative business models (Drupsteen & Wakkee, 2024; Konietzko et al., 2023). These efforts include innovations in sourcing strategies, brand partnerships, and materials research.

In particular, sourcing strategies are shaped by a diverse array of financial arrangements, including donations, payments for services, venture capital, raw material purchases, compensation for litter collection, and other nuanced forms of value exchange. For example, Case Study 2, commercial fisheries deposit their nets to the recycler, saving them tipping fees for traditional waste management. The money saved is instead donated to local nonprofits that “implement community projects in their area”. This is seen as a “net positive result” for the environment and communities, helping to manage a “once harmful waste” while generating social benefits. These financial incentives support the creation of new value networks that recover and reprocess materials into consumer-facing products, generating ecological, economic, and social benefits for communities affected by marine plastic pollution. Materials once considered waste are revalued through economic exchanges between remanufacturers, brands, and end-users.

### Branding and positioning

Interviewees in both Case Study 3 and Case Study 4 indicated that customer-facing brands derive significant value from incorporating materials sourced through regenerative supply networks. Unlike traditional supply chains, where incentives and pressures typically flow downstream (from brands to manufacturers and then to suppliers), RVNs reconfigure these relationships. They foster closer alignment between regenerative material suppliers, informal waste collectors, and brand identity.

These cases move beyond the principles of circularity by emphasizing transparency and place-based traceability within their sustainability narratives. Framing regeneration as a dimension of circularity (Ellen MacArthur Foundation, 2015) supports Tura et al.’s (2019) proposition that the circular economy can enhance brand value, serving as a strategic asset for sustainable branding and customer engagement. This is particularly evident in marine plastic entrepreneurship where product development and customer relationships are closely intertwined (Dijkstra & Planko, 2023).

### Governance and accountability

Despite the emergence of RVNs worldwide, there is currently no centralized coordination, institutional support, or overarching governance structure to guide their development. Several organizations such as Ocean Cycle, the Ocean Bound Plastic Certification, and Control Union Global are working to certify ocean and ocean-bound plastics. However, validating regenerative practices at the material level remains challenging. As found in our cases, many remanufactured materials central to these networks are trademarked as a means of communicating authenticity with customers. While trademarks play a key role in brand recognition, consumer trust, advocacy, and business growth, they lack the credibility and accountability of independent third-party certifications.

The proliferation of brands, labels, and certification schemes may also confuse consumers and create space for greenwashing (Dijkstra et al., 2022). Although entrepreneurs in this field are committed to transparency and accountability, conducting thorough impact assessments remains logistically unfeasible while simultaneously managing business operations. Only Case Studies 4 and 5 conducted detailed life cycle assessments comparing regenerated nylon with petroleum-based alternatives, highlighting the need for government or third-party support in quantifying and verifying impacts across the sector.

### Scaling and systems change

In line with previous research, the case studies and value maps reveal diverse strategies employed to meet material, economic, social, and ecological objectives within modest, regionally scaled initiatives. Brehmer et al. (2018) introduces the concept of *boundary spanning* to illustrate how various value exchange models such as partnerships, licensing, and platform strategies can amplify the impact of sustainable enterprises. In this study, the cases prioritized partnerships, certifications, and marketplace development to facilitate scale.

Case Study 1 pursued a relatively independent approach, investing in R&D and material innovation to expand its sustainable offerings and engage more broadly in social systems. Case Study 2 focused on building partnerships across both supply and demand, working closely with government agencies and private fishing operators to develop collection strategies. Case Studies 3 and 4 adopted marketplace models, selling certified proprietary materials to brand partners, which enabled them to increase collection efforts and deliver social value. Case Study 5 entered a corporate manufacturing partnership to co-develop a recycled material and broaden its fishing net sourcing. These examples underscore the importance of viewing scaling not solely as a business function, but as a multi-level socioecological process.

Policy frameworks ranging from local cleanup initiatives to international regulations are beginning to address plastic pollution and support remediation. As international efforts such as the UN Plastics Treaty negotiations gain momentum, it is essential to understand and support bottom-up regenerative models already in practice. These grassroots systems must be enabled, not hindered, by new policy structures. Current treaty debates include calls from ambitious states and organizations for upstream solutions, such as reducing plastic production and designing for reuse and recyclability which are generally more cost-effective and environmentally sound. However, vast quantities of legacy plastic already pollute terrestrial and marine environments and projections indicate continued growth in this waste stream (Lau et al., 2020). Recovering this plastic from beaches, waterways, and oceans is technically challenging and more expensive than upstream interventions (Hohn et al., 2020). Nevertheless, RVNs that successfully recover ocean plastic while generating social and economic benefits will be essential to restoring common ecological systems. The insights and strategies developed in these networks can serve as models for addressing other pollution challenges within the global commons.

### Practical implications

Emerging policy mechanisms aimed at authenticating materials derived from ocean plastic cleanup efforts are still in early stages with outcomes yet to be realized. The RVN framework breaks down vague and arbitrary descriptions of value into clear, distinct value exchange elements: material, social, ecological, and economic. This framework can serve as a foundational model to define how regenerative business claims are qualified, particularly in the context of marine debris recovery and global plastics governance. RVN would benefit from multi-stakeholder certification systems and incentive structures that standardize the value exchange process, redefining waste as a raw material, thus generating material regenerative value.

While the entrepreneurs and intraprenuers in these cases act as pioneers in regenerative supply networks, enabling structures for transnational reciprocity (Gualandris et al., 2024) remain underdeveloped. For example, in Case Study 4, a key operational challenge involved cross-border transportation of waste fishing nets. When shipped across national lines, these materials were often treated as waste and incurred import fees, rather than as raw materials, unlike petroleum which continues to benefit from significant subsidies for fuel and virgin plastic production. At the same time, global trade in recyclable materials has been restricted in recent years due to concerns over low-quality inputs. For instance, one interviewee described “Each country has different regulations around [the form of the material]. We might have to do an extra processing step that not might not even add any value to the actual recycling process, but we have to do it just to appease the customs in order to import the material into the country. If it’s in the category of scrap, it has to be within a certain size. You couldn’t send a complete fishing net. And so that’s been causing a huge hindrance on our ability to move freely from country to country the materials we’re collecting and also adding another expensive margin to the cost of our material”. Though some policy efforts have aimed to incentivize international recycling, the broader regulatory framework still poses barriers to the scaling of RVNs.

As the sourcing and market for remanufactured plastics becomes increasingly complex, governance mechanisms such as private certifications and public policies are emerging to increase transparency, deliver social benefits, and guard against greenwashing. For instance, an interviewee recounted how fishing nets in Panama are managed: “If you’re a fisherman in Panama, if you want to go and buy a new fishing net, you have to present a certified ticket that says my previous fishing net was responsibly disposed of. And that can only be issued by a certified waste management company that can handle that type of that type of waste.” This level of oversight and certification provides a greater level of integrity and transparency throughout the life cycle of the material.

Paradoxically, the proliferation of certifications, standards, and regulations can also create confusion among brands and consumers. If marine plastics follow the pattern of other sustainable materials, a few dominant certifications are likely to emerge as industry standards over time. Consistent with prior research (e.g., Dijkstra et al., 2021, 2022; Grassin & Dijkstra, 2024; Meyer, 2020; Zucchella & Previtali, 2019), this study reveals how multi-stakeholder networks enable the re-commercialization of polluting materials in the global commons by applying regenerative principles.

Another recurrent theme among interviewees was the difficulty of competing with the low cost of heavily subsidized petroleum-based raw materials. Collecting marine plastics through informal networks and volunteers incurs high operational costs, and the infrastructure needed to manage the reverse logistics of dispersed waste lacks the efficiency and scale required for cost competitiveness. One interviewee explained, “If I fill up a container of nets, it’s worth not $10,000 so […] the value of your materials doesn’t cover the costs, so you have to get that money from elsewhere”. In contrast, the petroleum industry has benefited from decades of aligned investment across labor, technology, and political frameworks. Many regenerative ventures report difficulty sustaining operations without supplementary revenue streams or policy mandates that increase demand (for example, minimum recycled content requirements in packaging). One proposed model is a standardized credit system in Asia, where much of the world’s plastic waste is concentrated and opportunities for remanufacturing are significant (Lee, 2021) Figs. 1–5.

While members of RVNs often share goals such as inclusion of marginalized stakeholders (Taylor & Rosca, 2023), marine ecosystem restoration, and sustainable production-consumption systems, there is a notable absence of shared industry organization, political advocacy, or communication platforms (Dijkstra et al., 2022). In some of the cases, minor competition and internal conflicts emerged among actors operating in different regions or developing ocean-bound plastic certifications and supply chains. For RVNs to play a meaningful role in addressing marine plastic pollution, they must articulate and promote alternative forms of value beyond financial metrics that are recognized and incentivized through supportive mechanisms. Entrepreneurs aiming to develop regenerative business models must understand principles of ecological and social restoration, cultivate skills in policy negotiation, and support customers with branding narratives that communicate the specific origin and impact of regenerative materials, all responsibilities that extend well beyond those required in conventional sourcing models.

Importantly, entrepreneurs and intrapreneurs often lack the time and financial capacity to engage in activities beyond their core business functions. In RVNs, the diversity of stakeholders, materials flows, and operational processes requires advanced levels of coordination and collaboration for basic daily functions of the organization. Industry-wide coordination should therefore be led by governmental or corporate entities with broader oversight and resources to ensure that regenerative practices are transparent, accountable, and consistent. All stakeholders, including customers seeking more ecologically-sound products, would benefit from greater oversight and clearer communication about regenerative practices and claims.

### Limitations

The study was limited in terms of its scope of analysis with a small number of cases, with many other cases of ocean plastic entrepreneurship (e.g., Dijkstra et al., 2021) and regenerative business models (Drupsteen & Wakkee, 2024; Konietzko et al., 2023) having been identified in recent years. While the current research was focused analyzing value exchange networks, there are many opportunities to expand on the quantitative and qualitative impacts of the growing number of organizations building RVNs with this and other polluting materials.

Furthermore, this study must be considered within the context of the immense scale of the plastic pollution crisis and the need for immediate action to remediate it. Given technological lock-in and socio-political drivers, the transition away from plastics will likely require decades of innovation and long term pollution remediation strategies must remain a high priority. However, re-integrating polluting plastics into value networks should not be viewed as a singular solution. Vigilance is necessary to ensure that regenerative business models do not inadvertently incentivize more plastic production or other forms of perverse incentives in plastic supply chains. The development of RVNs are not intended to replace much needed transitions in systems of production and consumption towards less polluting materials which is concurrently urgent and necessary.

### Future research

Future research should include quantitative measures of the various categories of regenerative impact in the context of the global commons’ pollution to create a more nuanced understanding of value beyond superficial economic measures. There is also a need to describe and document the less tangible forms of value (e.g. awareness raising, regulatory influence) created within RVNs that have yet to be analyzed.

While sustainability certifications have been well-documented, the area of certifications amongst RVN, which can validate the impact of social, material or ecological value exchange is fertile ground for exploration. As a regular theme in the studied cases, it is clear that further investigation is necessary.

Additionally, these cases provide insights into the overall market-forces and governance of pollutants that span international boundaries. Carbon, for example, which has led to numerous credit and marketplace schemes in an effort to curb and invest in emission reductions and remediations, may benefit from a similar value network analysis. Carbon markets also require similar substantiation in the consumer product market to identify and encourage new forms of value creation. Certifications of positive carbon impacts and co-benefits (e.g. biodiversity) may also be influential in the development of new materials. Similar case studies in carbon-sequestering materials are emerging as market and policy mechanisms incentivize technological development. Though carbon will require different mechanisms of collection and remanufacturing, there are many similarities with its place in the global commons. The need for certifications, complex RVNs, and trademarked materials will also likely emerge on a similar trajectory to the cases in this study.

## Conclusion

In summary, governance systems of the commons have evolved immensely in this globalized age with many new and long-standing examples of collective governance systems which divert the tragedy into shared benefit (Ostrom, 1990). RVNs constitute an emergent form of decentralized, voluntary governance that offers a partial but pragmatic approach to managing shared environmental resources. While not yet fully developed, viewing regenerative value as a series of exchanges (material, economic, ecological and social) may provide insights into business activities that is better aligned with regenerative theory. Though predominantly led by customer-facing brands and the environmentally-engaged customers that demand greater levels of corporate responsibility, an opportunity exists to institutionalize this framework through international governmental and nongovernmental organizations. Although governments are slow to align on major ecological, economic, material, and social agendas, this form of organizing for emergent forms of value across transnational boundaries to manage waste in the global commons demonstrates one role that the private sector entities can have in mitigating the current socioecological crises.

## Data Availability

Interview data is provided upon request by emailing the corresponding author.
